# Evaluation of hepatic lesions in 30 proven cases of MEN-syndrome

**DOI:** 10.1186/1470-7330-14-S1-P15

**Published:** 2014-10-09

**Authors:** N Fard, B Jobke

**Affiliations:** 1 German Cancer Research Center, Heidelberg, Germany

## Aim

MEN syndrome is one of the most important familial causes of neuroendocrine tumours. Radiological screening and follow-up for syndrome-related tumours play an important role in the life-long surveillance. Liver metastases (LM) from neuroendocrine tumours are common (75%) and significantly reduce the prognosis. Characterisation of liver lesions in these patients is a challenge for radiologists as LM are difficult to differentiate from benign liver lesions such as of haemangioma. In this study we aimed to give an overview of the radiological presentation of LM in MEN patients by different imaging modalities.

## Methods

We retrospectively evaluated 30 genetically-proven cases of MEN syndrome (type 1+ 2), who had a hepatic lesion. The findings of contrast-enhanced computer tomography (CE-CT) as well as ultrasound (US) were considered for the main diagnosis. The findings of other imaging techniques such as contrast-enhanced ultrasound (CEUS), magnetic resonance imaging (MRI) as well as PET-CT were evaluated and compared individually.

## Results

The most common liver lesions were haemangioma (40%) followed by LM (30%). The radiological findings in LM varied from hypodense lesions with low marginal contrast-enhancement to disseminated calcified lesions. The most common CT finding in early stage LM was hyperarterialised lesion with marginal contrast-enhancement in the portal venous phase as well as hyperechogenic appearance in US which mainly mimic the classic haemangioma.

## Conclusion

Better understanding of the radiological appearance of LM in patients with MEN syndrome will help in early tumour-detection and adjusted treatment. For this purpose multimodal imaging, primarily CE-CT in addition to CEUS or CE-MRI are essential for screening as well as follow-up controls.

